# Inconsistency between Human Observation and Deep Learning Models: Assessing Validity of Postmortem Computed Tomography Diagnosis of Drowning

**DOI:** 10.1007/s10278-024-00974-6

**Published:** 2024-02-09

**Authors:** Yuwen Zeng, Xiaoyong Zhang, Jiaoyang Wang, Akihito Usui, Kei Ichiji, Ivo Bukovsky, Shuoyan Chou, Masato Funayama, Noriyasu Homma

**Affiliations:** 1 Department of Radiological Imaging and Informatics, Tohoku University Graduate School of Medicine, Sendai, Japan; 2 National Institute of Technology, Sendai College, Sendai, Japan; 3 Department of Intelligent Biomedical System Engineering, Graduate School of Biomedical Engineering, Tohoku University, Sendai, Japan; 4 Faculty of Science, University of South Bohemia in Ceske Budejovice, Ceske Budejovice, Czech Republic; 5 Mechanical Engineering, Czech Technical University in Prague, Prague, Czech Republic; 6 Department of Industrial Management, National Taiwan University of Science and Technology, Taipei, Taiwan

**Keywords:** Deep learning, Validity assessment, Computer-aided diagnosis, Postmortem computed tomography, Drowning

## Abstract

Drowning diagnosis is a complicated process in the autopsy, even with the assistance of autopsy imaging and the on-site information from where the body was found. Previous studies have developed well-performed deep learning (DL) models for drowning diagnosis. However, the validity of the DL models was not assessed, raising doubts about whether the learned features accurately represented the medical findings observed by human experts. In this paper, we assessed the medical validity of DL models that had achieved high classification performance for drowning diagnosis. This retrospective study included autopsy cases aged 8–91 years who underwent postmortem computed tomography between 2012 and 2021 (153 drowning and 160 non-drowning cases). We first trained three deep learning models from a previous work and generated saliency maps that highlight important features in the input. To assess the validity of models, pixel-level annotations were created by four radiological technologists and further quantitatively compared with the saliency maps. All the three models demonstrated high classification performance with areas under the receiver operating characteristic curves of 0.94, 0.97, and 0.98, respectively. On the other hand, the assessment results revealed unexpected inconsistency between annotations and models’ saliency maps. In fact, each model had, respectively, around 30%, 40%, and 80% of irrelevant areas in the saliency maps, suggesting the predictions of the DL models might be unreliable. The result alerts us in the careful assessment of DL tools, even those with high classification performance.

## Introduction

Drowning is one of the leading causes of unnatural death worldwide, according to the World Health Organization [1]. In forensic medicine, drowning diagnosis via autopsy is difficult because of its nonspecific pathophysiology [2, 3]. The difficulties mainly arise from the absence of a single pathognomonic sign or test. Drowning may present a wide range of clinical and radiological manifestations, further complicated by variables such as water conditions, drowning scenarios, and the postmortem interval. Thus, it is crucial to notice that drowning diagnosis is based on sum of all the nonspecific findings and exclusion of other causes of death.

Autopsy imaging, such as postmortem computed tomography (PMCT), has been introduced to aid in the drowning diagnosis [4]. However, there is a shortage of forensic pathologists who can interpret imaging scans. Considering the above challenges, deep learning-based computer-aided diagnosis systems have been developed to assist forensic pathologists. Previous studies [5–7] have demonstrated the feasibility of deep learning (DL) models for drowning diagnosis using PMCT. These models have achieved high classification accuracy, but the validity of the models remains unknown. Certain research has raised concerns about the potential unreliability of DL models. For instance, some DL-based classification models for COVID-19 screening might generate predictions founded on irrelevant image features sourced from different data sources [8].

To prove the validity of the models, most studies [9–13] have made efforts to provide visual explanations for model predictions using visualization methods like Grad-CAM [14]. Although such visualization can provide insight into the attention of a model by highlighting the important areas on the input images [15, 16], these studies only use it for visual verification and did not carry out any assessment. Some studies had assessed the visual explanations of classification models with the ground truth, but they only conducted hit-or-miss evaluation [17, 18]. Given the inherent black-box nature of DL models, it becomes crucial to assess the validity of these models, which is to determine whether the areas to which the model assigns attention (which form the basis for its predictions) align with human observations.

In this study, we evaluated the validity of DL models for drowning diagnosis by examining the consistency level between model attention (saliency maps) and human observation (manual annotations). Three DL models that had reached the state of the art in a related work of drowning diagnosis [17] were first trained on an in-house PMCT dataset to generate saliency maps. Four annotators independently annotated lung features related to drowning on the dataset. The saliency maps and annotations were then compared and analyzed. Finally, we discussed potential problems and future work.

## Materials and Methods

The review board of our institute approved this retrospective study and waived the requirement for informed consent (IRB No. 2021–1-495).

### Study Sample and Scan Protocol

**Fig. 1:**
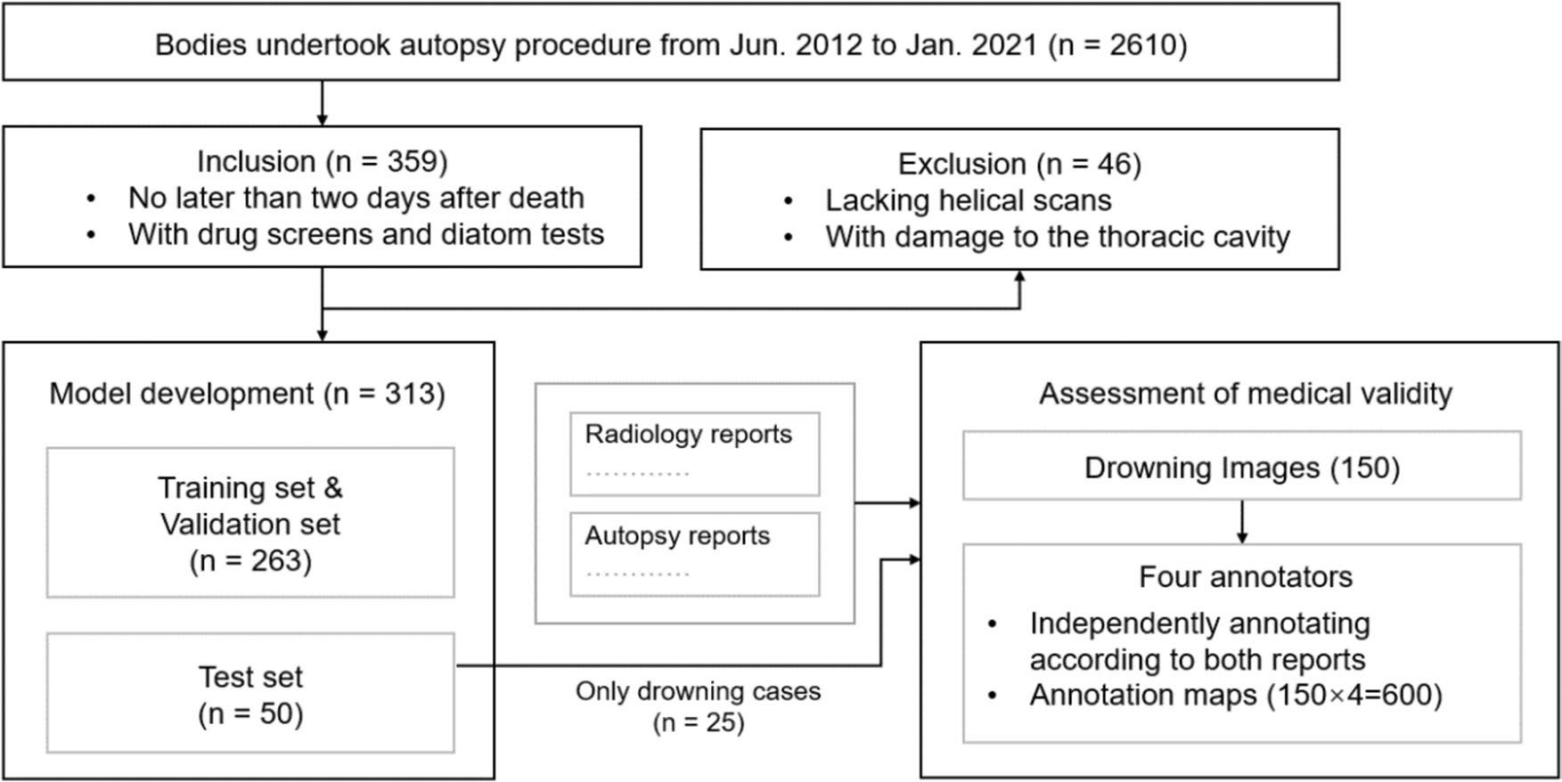
Case inclusion and exclusion of the training and test for the deep learning model development and the annotation

There were 2610 bodies that underwent pre-autopsy screening and autopsy at the study institution at Tohoku University from June 2012 to January 2021. Cases that were no later than 2 days after death and underwent drug screens and diatom tests were eligible for inclusion (*n* = 359). We further excluded 46 cases that were without helical scan or with damage to the thoracic cavity and obtained a study sample of 313 cases, including 153 drowning cases and 160 non-drowning cases, as shown in Fig. 1. Causes of non-drowning death include cardiovascular disease (*n* = 54), asphyxia other than drowning (*n* = 19), infection (*n* = 16), intoxication (*n* = 14), trauma (*n* = 14), alcoholic and diabetic ketoacidosis (*n* = 13), and others (*n* = 30).

The autopsy diagnosis was based on a comprehensive assessment including PMCT, on-site police investigation, and forensic autopsy. Each case (anonymized) had a free-text autopsy report from a forensic pathologist with 35 years of experience and a free-text radiology report from a board-certified radiologist with 20 years of experience (and 14 years of PMCT interpretation experience) and a radiological technologist who had 14 years of PMCT imaging experience. Both reports had achieved an agreement in the final diagnosis. Due to the particularity of autopsy images, we did not conduct external validation because it is difficult to obtain reliable dissection-proved autopsy imaging data.

PMCT scanning was performed on a multi-channel scanner (Canon Medical Systems, Japan). We obtained high-resolution (HR) chest scans of the lung with protocol of 135 kVp, 190–250 mAs, M-sized field of view (FOV), and 1.0-mm slices (size 512 × 512 pixels) every 30 mm through the chest in a four-row multi-slice mode and processed with lung kernel settings. Following the HR-CT scanning, volumetric helical scans were obtained from the head to the proximal femurs at 120 kVp with variable mAs, a beam pitch of 0.875, LL-sized FOV, and 2.0-mm collimation. The volumetric data allowed reconstruction of whole lung images from 2.0-mm slices, also with lung kernel settings.

### Model Architecture and Training

The models are based on AlexNet [19], VGG16 [20], and Inception-ResNet-V2 (InResV2) [21] and have 4 M, 15 M, and 56 M parameters, respectively. The original layers on top of the last convolutional block of AlexNet and VGG16 were replaced by a global average pooling (GAP) layer, two smaller fully connected (FC) layers, and a softmax layer. The 313 PMCT cases were randomly split into training and test set at a ratio of around 85%:15%, and all models were trained from scratch. The loss function was binary cross-entropy, and the optimizer was Adam with a learning rate of 1e − 5 and a decay rate of 1e − 6. To determine whether the training was done, early stopping was applied if the validation loss was no longer decreasing in ten epochs.

**Fig. 2:**
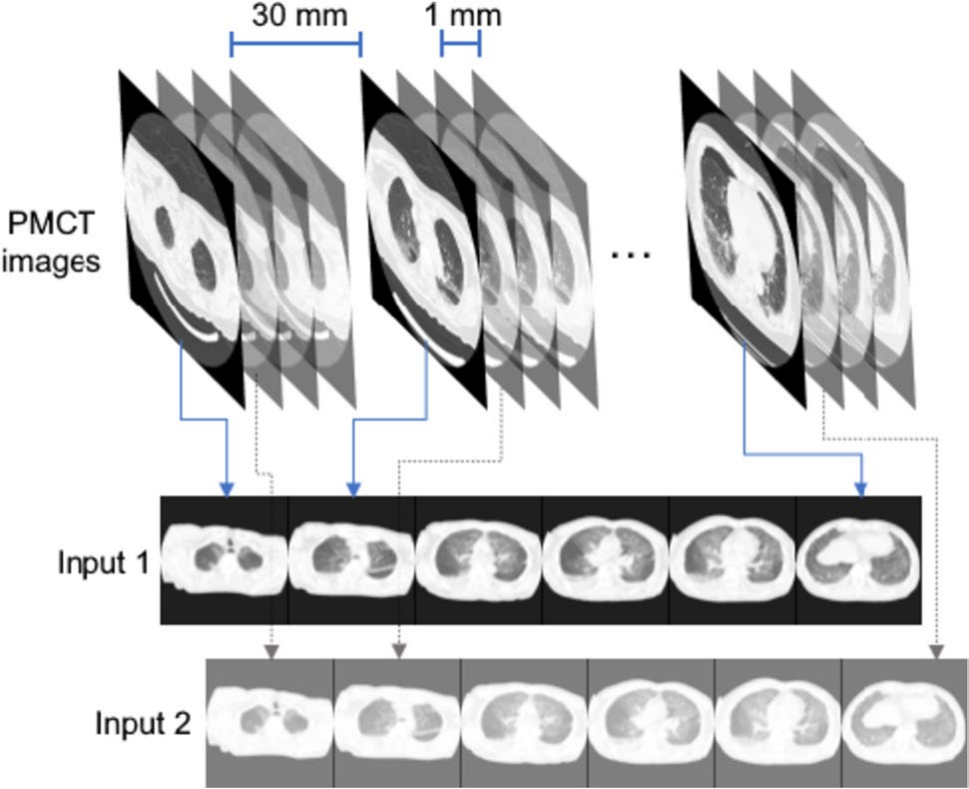
The input of the models [17]. PMCT images with the same interval were concatenated into one 2D image, which was used as an input of the models

In terms of the model input, we adopted the method mentioned in [17], which can produce better classification and visualization results. To be more specific, a single 2D image only offers information in the transverse plane, resulting in the loss of 3D anatomical details, while 3D volumetric input requires 3D models, resulting in much more parameters to be fitted. Since PMCT data were obtained with a protocol of a four-row multi-slice mode (four 1.0-mm slices every 30 mm), we can pick out images of a case with the same interval and concatenate them vertically or horizontally to embed 3D information into a 2D image, as shown in Fig. 2. The area under the receiver operating characteristic curve (AUC), accuracy, sensitivity, and specificity were used to evaluate the models.

### Saliency Maps

**Fig. 3:**
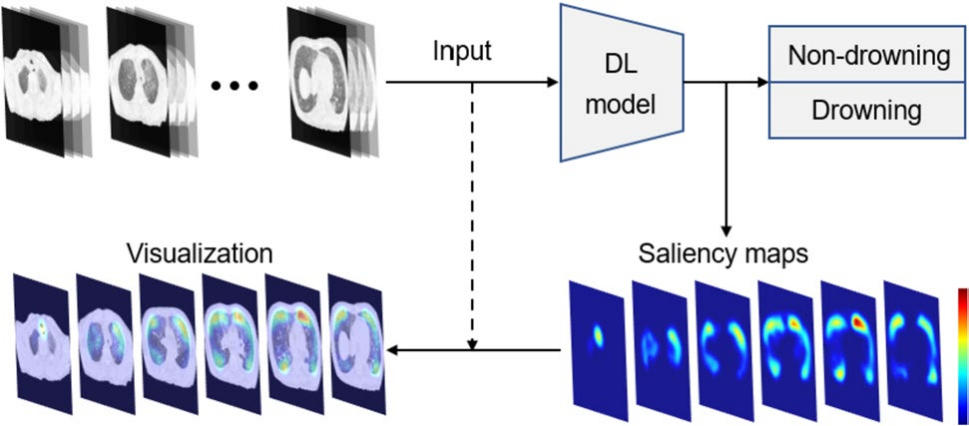
A simple illustration of a model and its visualization. Models take PMCT images as the input and classify them into non-drowning and drowning. Then, saliency maps are then calculated using the weights of models and further projected to the input to get visualization results. A warmer color in the saliency maps represents a stronger model attention

For most of the papers that generated visual explanation to model predictions, the major saliency methods can be summed up into two kinds: perturbation-based [22, 23] and gradient-based methods [14, 15, 24, 25]. Perturbation-based methods are intuitive and simple to implement but are also time consuming, and gradient-based methods could be of low quality and noisy or show false confidence in visualization results due to the global pooling operation and the gradient vanishing issue in models [16, 26]. Following this discover, Wang et al*.* proposed a novel gradient-free method named Score-CAM [27], which bridges the gap between perturbation-based and gradient-based methods. In this study, we adopted the Score-CAM to generate saliency maps and compared them with annotations, as shown in Fig. 3. A warmer color (red) in the saliency maps represents a stronger model attention (higher activation).

### Human Annotations

To evaluate the saliency maps, four annotators (radiological technologists with over 1 year of clinical experience) used an annotation tool (Labelme, v5.1.1) [28] to annotate 150 images selected at the same interval from the 25 drowning test cases, and finally, we obtained 600 annotation maps. To ensure the quality and validity of the annotations, we invited the experienced radiological technologist (14 years of PMCT imaging experience) as an annotation supervisor and made some rules. 

The rules are as below:

1. Annotate features that are associated with drowning according to the reports: consolidation and opacity (granular opacity, ground-glass opacity, edema-like opacity, etc.) in lungs, airway with fluid or solid (only above the carina of trachea), and pleural effusion. Note that we did not distinguish them in the evaluation because the models were for binary classification.
2. Follow both the autopsy and radiology reports and ensure coherency among continuous images.
3. Empty annotation is allowed if features are not significant.

The annotation process referred to the two-phase annotation process of a benchmark dataset called LIDC/IDRI [29] and was organized as below:

1. In the initial blinded-read phase, four annotators independently annotated PMCT scans. Then, the annotation supervisor blindly reviewed and gave comments on each annotation.
2. In the second unblinded-read phase, annotators independently revised their annotations according to the comments.

By following this two-phase process, we aimed to enhance the accuracy of the annotations. Instead of averaging or fusing annotations to achieve an agreement, we kept the differences to evaluate the DL models with the real-world variability of image interpretation.

### Validity Assessment

Intersection over union (IoU) was used to measure the consistency between annotations and saliency maps. Two IoU-derived metrics [30], namely Saliency Cover (SC) and Ground Truth Cover (GC), were used to describe the how saliency maps and annotations cover each other. Let *S* represent saliency maps and *G* represent annotations, then the metrics can be denoted as:

$$IoU=\frac{|S\cap G|}{|S\cup G|}, SC= \frac{|S\cap G|}{|S|}, GC= \frac{|S\cap G|}{|G|}$$

All metrics range from 0 to 1, with 1 indicating a perfect match between the predicted saliency map (S) and the ground truth (G). IoU, or Jaccard index, measures the overlap between S and G. SC assesses how much of S aligns with G, while GC evaluates how much of G is covered by S. In essence, SC reflects precision, representing the true saliency among all predictions, and GC implies sensitivity, indicating the true saliency among all ground truth annotations.

**Fig. 4:**
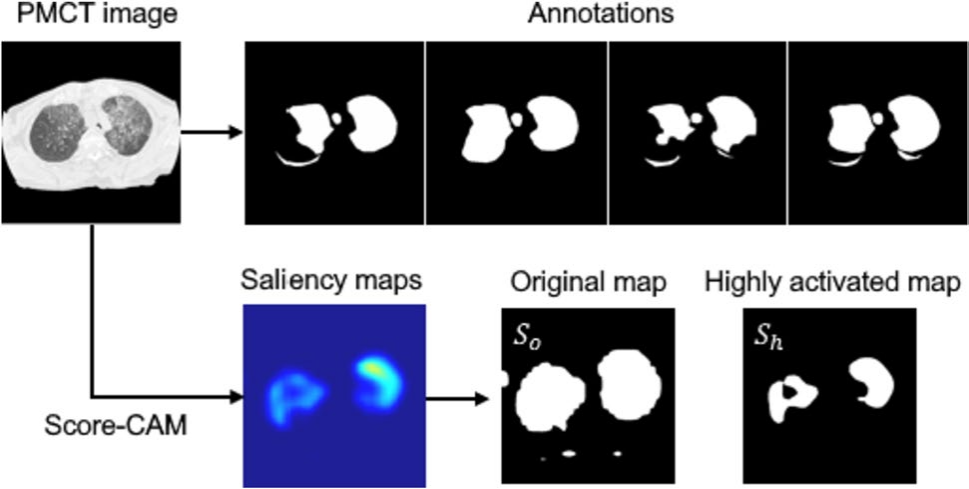
An example of the annotations and saliency maps used for validity assessment. Saliency maps were binarized into the original maps and highly activated maps to compare with the annotations. *S*_o_ = original maps, *S*_h_ = highly activated maps

To decide whether the saliency maps are consistent with annotation, we adopted a default threshold of 0.5 for IoU, as also used in many other benchmark tasks [31, 32]. An example of annotations and saliency maps are shown in Fig. 4. Annotations are binary masks, while saliency maps have continuous pixel values, so in addition to the original saliency maps *S*_*o*_, we applied Otsu thresholding [33] to obtain highly activated saliency maps *S*_*h*_ to see the discriminative areas.

### Statistics Analysis

Dunn’s test was used for comparison of two groups and Kruskal–Wallis *H* test for three or more groups, with *P* < 0.05 for significance. AUC, accuracy, sensitivity, and specificity of the models were described by means with 95% CIs using nonparametric bootstrapping. Considering the asymmetric distributions of IoU, SC, and GC, they were described by medians with interquartile ranges (IQRs). All analysis was performed using Python (v3.9.10, with SciPy v1.9.3 and scikit-learn v1.2.0; Python Software Foundation).

## Results

### Characteristics of the Study Sample

The study sample is described in Fig. 1 and Table 1. There were 359 cases from 2012 to 2021 that met our inclusion criteria; 46 were excluded due to lacking helical scans or having damage to the thoracic cavities. A total of 313 PMCT cases contained 153 drowning cases (128 for training and 25 for test) and 160 non-drowning cases (135 for training and 25 for test). There was no significant difference between the age of training set and test set according to Dunn’s test (*P* > 0.05).

**Table 1:** Baseline characteristics of the study sample

	Drowning		*P* value	Non-drowning		*P* value
Dataset	Training	Test		Training	Test	
Age^a^						
Male	61.28 ± 11.36	62.0 ± 12.87	0.85	55.17 ± 20.0	52 ± 14.12	0.14
Female	67.67 ± 14.93	73.18 ± 14.29	0.10	56.73 ± 22.58	63.33 ± 24.25	0.96
Sex*						
Male	80 (62.5)	14 (56)		84 (62.2)	16 (64)	
Female	48 (37.5)	11 (44)		51 (37.8)	9 (36)	
Total	128	25		135	25	

^a^Data are means ± standard deviations

*Data are numbers of participants, with percentages in parentheses

### Classification Performance of DL Models

**Fig. 5:**
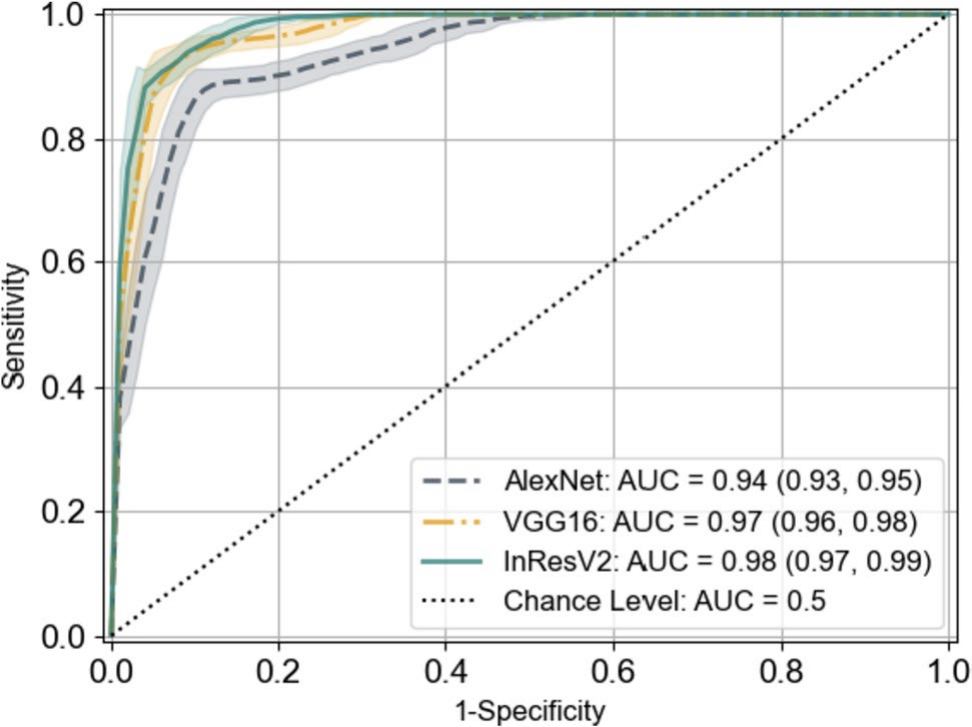
Receiver operating characteristic curves of the deep learning models with 95% CIs

The receiver operating characteristic (ROC) curves and the classification performance of models are summarized in Fig. 5 and Table 2. As can be observed, the three models had achieved high AUC, accuracy, sensitivity, and specificity. VGG16 achieved almost the same performance as InResV2, but with a simpler architecture and fewer parameters. This suggests that the improvement from using deeper models on small dataset is limited.

**Table 2:** Classification performance of the deep learning models

Model	AUC	Accuracy (%)	Sensitivity (%)	Specificity (%)
AlexNet	0.94 [0.93, 0.95]	88.9 [88.5, 89.4] (327, 368)	87.5 [86.9, 88.3] (161, 184)	90.2 [89.6, 90.9] (166, 184)
VGG16	0.97 [0.96, 0.98]	92.1 [91.6, 92.4] (338, 368)	95.7 [95.3, 96.1] (176, 184)	88.5 [87.8, 89.0] (163, 184)
InResV2	0.98 [0.97, 0.99]	92.1 [91.8, 92.5] (338, 368)	91.3 [90.7, 91.8] (168, 184)	92.9 [92.5, 93.4] (171, 184)

Data are the mean with 95% CI in brackets and the number of cases in parentheses. AUC = area under the receiver operating characteristic curve

### Human Annotations

Unlike risk assessment or diagnosis results, analyzing variability in annotations is challenging because such pixel-level annotations are unstructured data with nonuniform shapes, quantities, and positions. The intraobserver variability was not applicable because of the second unblinded-read phase in the annotation process. To show the interobserver variability among four annotators (A1–A4), we used the area of each annotation map as an index, which was simple but efficient. For the annotation area of each image, A2, A3, and A4 showed no difference (*P* = 0.20, Kruskal–Wallis *H* test), but they were significantly different from A1 (*P* < 0.001, Kruskal–Wallis *H* test). However, we did not exclude A1’s annotations because there is no specific criterion for the interobserver variability, especially for image interpretation [34]. In addition, the radiologists approved all annotations as “annotations made by different individuals may not be precise but could be correct.”

### Validity Assessment

IoU measures the consistency level between the human annotations and the saliency maps; SC and GC describe how much saliency and annotation was covered by each other, respectively. We used the overall medians of IoU, SC, and GC between four annotators and saliency maps in the following unless otherwise noted. 

Table 3 summarized the detailed statistical descriptions calculated on the original saliency maps *S*_o_. Both IoU and SC of A1 were significantly smaller than A2, A3, and A4 (all *P* < 0.001, Dunn’s test). AlexNet exhibited moderate consistency (IoU of 0.48, SC/GC of 0.66/0.67), with approximately 33% of human annotations not covered and 34% of the saliency maps containing irrelevant information. VGG16 displayed poor consistency with a high GC of 0.90 and low IoU/SC of 0.34/0.37, mainly as a superset of annotations, encompassing over 60% irrelevant content. InResV2 had low IoU/SC of 0.14/0.14, with a GC of 1 indicating complete annotation coverage but producing coarse-grained maps that extended to the entire annotation area.

**Table 3:** Consistency analysis of the original saliency map *S*_*o*_

Model	Metrics	Medians*	A1	A2	A3	A4
AlexNet	IoU	0.48	0.42 (0.34, 0.47)	0.49 (0.35, 0.55)	0.50 (0.37, 0.56)	0.52 (0.41, 0.58)
	SC	0.66	0.55 (0.43, 0.59)	0.67 (0.57, 0.70)	0.68 (0.62, 0.74)	0.72 (0.65, 0.79)
	GC	0.67	0.68 (0.63, 0.80)	0.67 (0.61, 0.77)	0.67 (0.61, 0.77)	0.67 (0.63, 0.77)
VGG16	IoU	0.34	0.29 (0.21, 0.34)	0.33 (0.26, 0.42)	0.37 (0.27, 0.43)	0.38 (0.29, 0.46)
	SC	0.37	0.31 (0.21, 0.36)	0.36 (0.27, 0.44)	0.39 (0.28, 0.46)	0.40 (0.31, 0.48)
	GC	0.90	0.90 (0.83, 0.95)	0.90 (0.82, 0.93)	0.90 (0.83, 0.93)	0.90 (0.84, 0.94)
InResV2	IoU	0.14	0.12 (0.09, 0.13)	0.14 (0.12, 0.16)	0.14 (0.12, 0.17)	0.15 (0.13, 0.17)
	SC	0.14	0.12 (0.10, 0.13)	0.14 (0.12, 0.16)	0.14 (0.12, 0.17)	0.15 (0.13, 0.17)
	GC	1	1 (1, 1)	1 (1, 1)	1 (1, 1)	1 (1, 1)

Except where indicated, data are medians with IQRs in parentheses

*A1–A4* Annotator1–Annotator4, *IoU* intersection over union, *SC* saliency cover, *GC* ground truth cover

*Data are overall median of each score on A1–A4

Similarly, Table 4 showed the results on the highly activated saliency maps *S*_h_. Both IoU and SC of A1 were significantly smaller than A2, A3, and A4 (all *P* < 0.001, Dunn’s test). AlexNet showed poor consistency with a high SC of 0.80 but low IoU/GC of 0.19/0.20, primarily representing a subset of annotations, covering only 20%. VGG16 also displayed poor consistency, with a decrease in GC (from 0.90 to 0.36) and an increase in SC (from 0.37 to 0.62), resulting in a slight IoU change (from 0.34 to 0.29) due to the removal of a substantial irrelevant portion after thresholding. InResV2 had a decrease in GC to 0.63 after thresholding and low IoU/SC of 0.17/0.19, indicating poor consistency in its highly activated saliency maps.

**Table 4:** Consistency analysis of the highly activated saliency map *S*_*h*_

Model	Metrics	Medians*	A1	A2	A3	A4
AlexNet	IoU	0.19	0.17 (0.13, 0.25)	0.19 (0.12, 0.22)	0.20 (0.12, 0.23)	0.18 (0.11, 0.24)
	SC	0.80	0.66 (0.55, 0.78)	0.82 (0.63, 0.88)	0.84 (0.70, 0.91)	0.88 (0.75, 0.95)
	GC	0.20	0.20 (0.13, 0.27)	0.21 (0.12, 0.24)	0.21 (0.12, 0.24)	0.18 (0.12, 0.24)
VGG16	IoU	0.29	0.24 (0.15, 0.33)	0.28 (0.20, 0.35)	0.31 (0.19, 0.36)	0.32 (0.22, 0.38)
	SC	0.62	0.50 (0.39, 0.62)	0.62 (0.46, 0.76)	0.67 (0.53, 0.77)	0.68 (0.59, 0.78)
	GC	0.36	0.36 (0.25, 0.48)	0.36 (0.24, 0.47)	0.37 (0.25, 0.46)	0.36 (0.26, 0.46)
InResV2	IoU	0.17	0.13 (0.10, 0.19)	0.16 (0.13, 0.21)	0.18 (0.14, 0.23)	0.20 (0.15, 0.24)
	SC	0.19	0.15 (0.11, 0.19)	0.18 (0.14, 0.23)	0.20 (0.15, 0.25)	0.22 (0.17, 0.26)
	GC	0.63	0.58 (0.54, 0.77)	0.64 (0.51, 0.78)	0.64 (0.55, 0.79)	0.64 (0.57, 0.78)

Except where indicated, data are medians with IQRs in parentheses

*A1–A4* Annotator1–Annotator4, *IoU* intersection over union, *SC* saliency cover, *GC* ground truth cover

*Data are overall median of each score on A1–A4

**Fig. 6:**
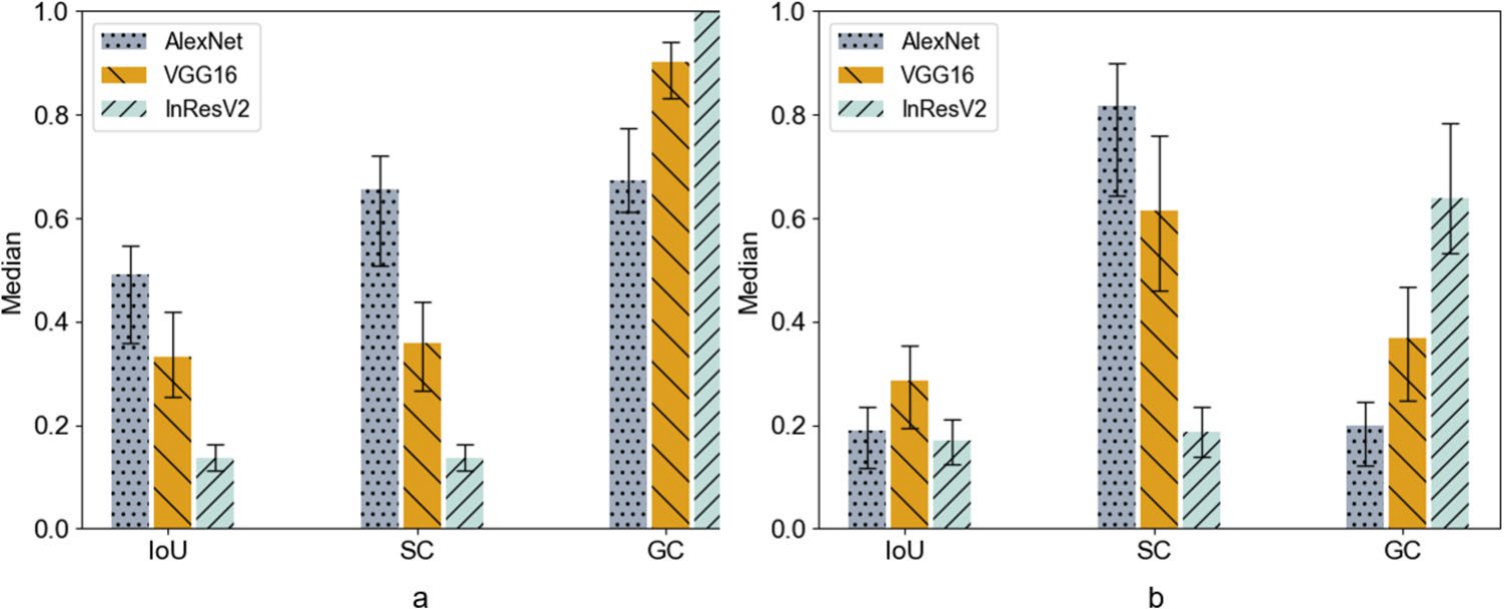
Averaged median IoU, SC, and GC of the original saliency maps *S*_*o*_ (**a**) and the highly activated saliency maps *S*_*h*_ (**b**). Error bars indicate the interquartile ranges

For a better view, we also presented the overall medians of the IoU, SC, and GC scores using bar plot. It can be observed that *S*_*o*_ of the models exhibited varying levels of consistency with the annotations in Fig. 6a. In the highly activated saliency maps *S*_*h*_ (Fig. 6b), there were shifts in the patterns, which was mainly caused by reduced irrelevant information after thresholding.

In summary, we found significant inconsistency between the human annotations and the saliency maps of the models. Additionally, models with higher classification performance showed more inconsistency, possibly due to overfitting on the small-scale dataset.

**Fig. 7:**
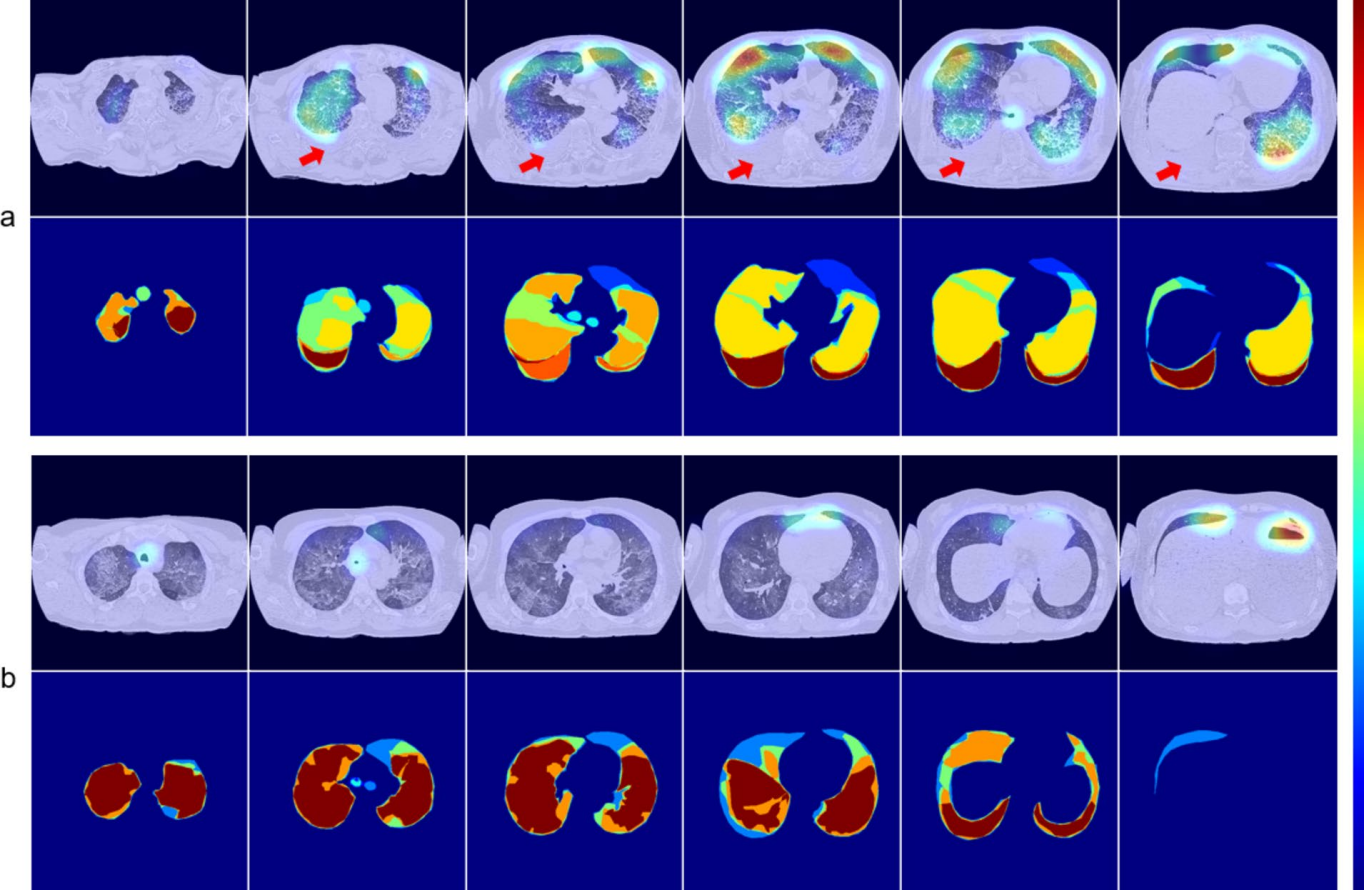
Examples of saliency maps (top rows) and corresponding overlapped annotations (bottom rows) are provided for a true positive case (**a**) and a false negative case (**b**). The red arrows highlight the region of pleural effusion that the model overlooked. Warmer colors indicate higher activation in the saliency maps and stronger agreement among annotators

To illustrate the inconsistency between model attention and human annotations, we showed a true positive cases and a false negative case of VGG16. In Fig. 7a, there is a notable inconsistency between the saliency maps and their corresponding annotations. While most annotators marked the areas with pleural effusion, the model did not effectively capture this feature, resulting in a lack of response. In the false negative case depicted in Fig. 7b, the model’s prediction was primarily based on the identification of dry airways and airway-like cavities, while it ignored the presence of fluid-filled airways and pleural effusion. This might be attributed to the fact that dry airways possess well-defined edges that can be more easily detected by computer vision, leading the model to regard them as feature-rich objects. In contrast, the fluid-filled airways and pleural effusion can resemble blood vessels and tissues and are therefore more difficult to distinguish. Furthermore, we observed variations among annotators’ interpretations: one annotator omitted the lung area, while all others included it in their annotations.

## Discussion

In this study, we trained three DL classification models for drowning diagnosis, and obtained high AUCs of 0.94 (95% CI: 0.93, 0.95), 0.97 (95% CI: 0.96, 0.98), and 0.98 (95% CI: 0.97, 0.99), respectively. To evaluate the validity of these models, we measured the consistency level between the saliency maps of models and human annotations. Unexpectedly, the best IoU scores were only 0.52, 0.38, and 0.20, respectively, with 30%, 40%, and 80% of the areas in the saliency maps being irrelevant according to the SC and GC scores. To our surprise, the models with higher classification performance had higher inconsistency compared to the human annotations, which may indicate overfitting due to the excessive parameters of the models.

One challenge we faced in this study is interobserver variability, which is especially prevalent in image interpretation tasks that rely on subjective perception. For example, when one person annotates a cat image, he or she may mark the cat’s face, while another may mark the entire profile, and both approaches may be considered correct. In medical imaging, where images may contain ambiguous features, this variability may be even more pronounced. Our results show that the total area of annotations from one of the annotators (A1) was significantly smaller than that of the other annotators (*P* < 0.001, Kruskal–Wallis *H* test), resulting in smaller scores of IoU and SC, as all metrics were calculated based on the area of annotations and saliency maps. This highlights the significant impact that ground truth annotations can have on the training and evaluation of models.

Some possible solutions to this challenge are as follows. First, keeping a detailed record of the annotation process could help identify and correct errors or biases that may have occurred. Second, having an expert supervise and review the annotation process can help improve the accuracy and quality of the annotations, as was employed in this study. Finally, crowdsourcing could be an effective approach to obtain high-quality annotations. For example, a collaborative framework [35] has been proposed to engage medical students and pathologists in producing quality labels for cell nuclei, without sacrificing diversity. By collecting a larger number of annotations from multiple annotators, we can mitigate the bias and variability in the training and evaluation of our models.

There are some limitations in this study. First, we could not find a forensic pathologist who can interpret radiological images to make annotations directly, which is one of the motivations for developing the CAD system. To ensure the credibility of the annotations, we adopted a two-phase annotation process, referred to the autopsy and radiology reports during annotation, and revised the annotations according to the comments of the experienced radiologist. The second issue is an inherent problem in the evaluation method. We used IoU and its derivatives SC and GC to measure the similarity and inclusive relation between annotations and saliency maps. IoU is a common and suitable metric for detection and segmentation tasks because the outputs of models, e.g., bounding boxes and segmented maps, are designed to be as close as possible to the annotations. However, the original outputs of a classification model are numbers corresponding to numerical labels, not annotation maps, which leads to a significant difference in area between saliency maps and annotations. In fact, the area of *S*_*o*_ was 2.92 to 3.97 times that of the annotations, while *S*_*h*_ was only 0.60 to 0.82 times that of the annotations. Consequently, even if all saliency maps and annotations overlap perfectly, we can only have a maximum IoU score of around 0.35 for *S*_*o*_ and 0.82 for *S*_*h*_, not 1, so do the SC and GC. Although it seems improper to evaluate classification models using these metrics, there is no criterion for such evaluation, especially for image interpretation.

To address these problems, the validity of classification models needs further research, possibly by combining deep learning with detailed human expertise, as is common in detection/segmentation tasks. It is worth noting that interobserver variability can be observed among annotators, which may affect the evaluation results. Therefore, the annotation process and evaluation metrics should be carefully considered. Our future work will focus on developing deeply supervised methods to improve the validity of our models and alleviate the negative impact of interobserver variability on evaluation results.

## Conclusion

Three DL models were trained for drowning diagnosis and achieved high classification performance. However, the saliency maps generated from these models showed inconsistency attention with human annotations, suggesting that the models may be unreliable from the perspective of medical image diagnosis, or the annotations were subjective and prone to biases. The result alerts us in the careful assessment of DL tools, even those with high classification performance.

## Data Availability

Data are accessible from the corresponding author by request, subject to approval from the institutional review board.
